# Evaluation of a nanosecond dissipative soliton resonance fiber laser for multiphoton microscopy

**DOI:** 10.1038/s41598-025-28372-0

**Published:** 2025-12-18

**Authors:** Katarzyna Kunio, Piotr Bojęś, Grzegorz Soboń, Karol Krzempek, Jakub Bogusławski

**Affiliations:** Laser & Fiber Electronics Group, Faculty of Electronics, Photonics and Microsystems, Wrocław University of Science and Technology, wyb. S. Wyspiańskiego 27, 50-370 Wrocław, Poland

**Keywords:** Multiphoton microscopy, Dissipative soliton resonance, Fiber lasers, Nanosecond laser pulses, Optics and photonics, Physics

## Abstract

Multiphoton microscopy often relies on femtosecond lasers due to their high peak powers and short pulse durations. However, these systems (e.g., Ti:Sapphire) are expensive, complex, and require dispersion compensation to maintain pulse parameters in the sample plane. Nanosecond sources, such as dissipative soliton resonance (DSR) lasers, offer a compact, low-cost, fully fiber-based alternative that eliminates the need for dispersion management due to their longer pulse durations. Here, we demonstrate the use of a DSR laser for multiphoton imaging. The system delivers 1064 nm nanosecond pulses at a 2 MHz repetition rate. We show that high-quality images can be obtained using a non-complex laser source by optimizing the peak and average power of the DSR laser pulses. Comparative experiments with a femtosecond laser demonstrate that similar fluorescence levels can be achieved from the same sample, while also highlighting the strong impact of dispersion on femtosecond pulses compared to the robustness of nanosecond pulses. We present the first demonstration of the application of a DSR laser in multiphoton microscopy, showing that it is a viable alternative for more complex sources, expanding access to the technique in less demanding environments.

## Introduction

Multiphoton microscopy^1–3^ is a nonlinear imaging technique that enables high-resolution, three-dimensional imaging deep within biological tissues^4,5^ with minimal photodamage^6^. Unlike single-photon excitation, which illuminates the entire light path and often results in out-of-focus fluorescence, the multiphoton process requires the near-simultaneous absorption of two or more low-energy photons, restricting fluorescence emission to the focal point^7,8^. This spatial confinement significantly reduces the background signal and limits photobleaching and phototoxicity outside the imaging plane. Furthermore, the use of near-infrared excitation wavelengths enhances tissue penetration, allowing for imaging at depths of several hundred micrometers in scattering samples^5,9^. These attributes make multiphoton microscopy particularly well-suited for in vivo and long-term imaging applications^10–12^, where maintaining tissue integrity is critical. As such, it has become an essential tool across many disciplines, including neuroscience^13,14^, ophthalmology^15,16^, developmental biology^17,18^, and immunology^19,20^, where non-invasive, deep-tissue imaging is required.

Traditionally, the multiphoton process requires ultrashort laser pulses with high peak power to achieve sufficient nonlinear excitation^21^. Because of this, the most commonly employed light sources in multiphoton microscopy systems are Ti:Sapphire^22,23^ and femtosecond fiber lasers^21,24^. These sources generate femtosecond pulses at repetition rates of tens of megahertz, typically within the 650–1180 nm spectral window^25^. Ti:Sapphire lasers deliver broad tunability and superior pulse quality, yet their high cost, operational complexity, and maintenance challenges present significant barriers to widespread use. Femtosecond fiber lasers provide a more compact and robust alternative, though they often sacrifice tunability and can be limited in peak power or pulse energy^26^. The reliance on ultrashort laser pulses has somewhat constrained the range of potential laser sources considered suitable for multiphoton microscopy. Regardless of the laser type, achieving ultrashort pulses in the sample plane requires the use of bulk-optic pulse compressors. Femtosecond pulses are very susceptible to chromatic dispersion effects of the microscope’s components, which need to be precisely compensated. This, in practice, proves to be challenging for end-users of such systems (e.g., biologists or medical professionals). Moreover, any modification of the optical setup, including routine microscope objective replacement, necessitates a precise recalibration of the dispersion compensation system.

However, longer nanosecond pulses can also be used for the multiphoton process under appropriate conditions^27^. In particular, DSR fiber lasers^28,29^ offer a promising alternative to traditionally used laser sources. DSR lasers produce high-energy nanosecond pulses at megahertz-level repetition rates while maintaining a simple, robust architecture. Their small physical dimensions and inexpensive fiber-based design make them appealing for compact and cost-effective multiphoton imaging systems. Nanosecond pulses are significantly less susceptible to dispersive effects than the usually used femtosecond pulses, making them an attractive option for use in endoscopic applications^30^. Moreover, while the peak power of nanosecond pulses is lower compared to femtosecond sources, their higher pulse energy can compensate for it by sustaining multiphoton excitation at the focal plane. This makes them a good choice for use in imaging systems where parameters such as resolution, photodamage thresholds, and excitation efficiency must be carefully balanced.

The two-photon fluorescence signal intensity during imaging can be controlled by the interplay between the average power at the sample plane (*P*_*avg*_), the pulse peak power (*P*_*peak*_), the pulse duration (*τ*_*p*_), and the repetition rate (*f*_*rep*_) of the laser source:

1 $$n\sim \frac{{P}_{avg}^{2}}{{\tau }_{p}\cdot {f}_{rep}}={P}_{peak}^{2}\cdot {\tau }_{p}\cdot {f}_{rep},$$

where *n* is the average number of photons emitted per second by the fluorescing medium. Thanks to this relationship, we can adjust the parameters of the DSR laser accordingly to accommodate its long pulse duration for use in multiphoton microscopy. The DSR laser naturally operates at a low repetition rate, which is advantageous for multiphoton microscopy as it partially compensates for the longer pulse duration. The remaining compensation can be achieved by increasing the average power at the sample. The two-photon fluorescence signal intensity can also be understood by considering the response to a single laser pulse (*n*_*p*_), which is proportional to the integral of squared instantaneous power *P(t)*^31,32^:

2 $${n}_{p}\propto C\cdot \underset{0}{\overset{{t}_{exp}}{\int }}{P}^{2}\left(t\right)dt$$

where *C* is a factor describing all process efficiency parameters (e.g., two-photon absorption cross-section, quantum efficiency, and fluorophore concentration), and *t*_*exp*_ is the exposure time.

Here, we demonstrate the successful use of an all-fiber DSR laser in multiphoton microscopy, utilizing pulses with durations ranging from 1 to 4 ns at a repetition frequency of 2 MHz and a central wavelength of approximately 1064 nm. To our knowledge, this is the first demonstration of such a system in multiphoton microscopy applications. Additionally, we provide a comprehensive performance comparison between our nanosecond system and a femtosecond fiber laser, evaluating key parameters such as pulse energy, peak power, and multiphoton excitation efficiency.

## Methods

### Dissipative soliton resonance fiber laser

**Fig. 1:**
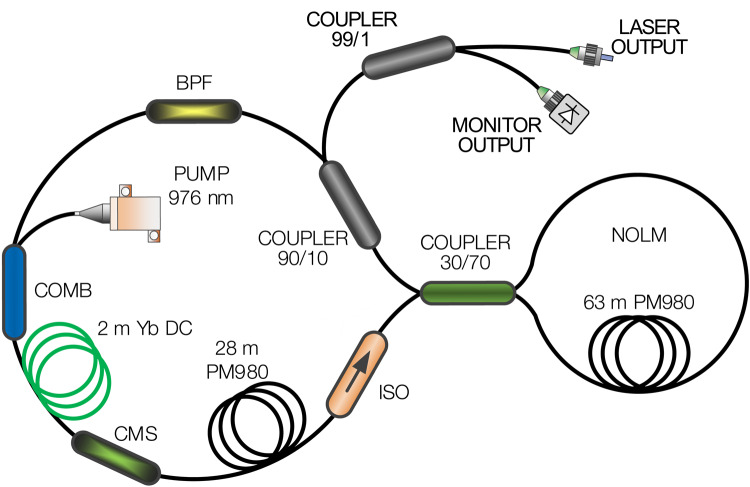
Schematic of the DSR fiber laser architecture. ISO–isolator, CMS–cladding mode stripper, Yb DC–ytterbium double-clad active fiber, COMB–pump/signal combiner, BPF–bandpass filter, NOLM–nonlinear optical loop mirror, PM980–polarization maintaining single mode fiber.

An all-fiber, DSR mode-locked laser in a nonlinear optical loop mirror (NOLM) configuration was used as the source of the nanosecond-long 1064 nm pulses (Fig. 1). The laser was designed in the figure-of-eight (F8L) scheme, and all used components were of the polarization-maintaining (PM) type, which provides a high degree of stability of the pulse output parameters and allows for easy and reproducible self-starting of DSR mode-locking. The unidirectional gain loop consisted of a 2 m double-clad ytterbium (Yb) active fiber (PLMA-YDF-10/125-HI-8, Nufern) pumped via a 976-nm multimode laser diode, a cladding mode stripper, a 2 nm bandpass filter, a spool of 28 m PM980 passive fiber, a 90:10% output coupler, and an isolator. The phase shift between the counter-propagating pulses, necessary to obtain DSR mode-locking in the NOLM configuration, was achieved by using a 70:30% coupler to connect the NOLM loop and the unidirectional loop. The net dispersion in the proposed cavity was at the level of 2.56 ps^2^, including 0.05 ps^2^ from double-clad active fiber and 2.51 ps^2^ from additional passive fiber spools and fiber components. The length of the passive fiber was adjusted to achieve a 2 MHz pulse repetition rate, chosen as an optimum balance between sufficient pulse energy for efficient multiphoton excitation and an adequate number of excitation pulses per pixel (~ 10) to support practical imaging speed without requiring complex synchronization. An additional 99/1% tap coupler was spliced to the output port of the laser. The 99% port was used to deliver the pulses to the multiphoton microscope, while the 1% port was used to monitor the pulse parameters using a fast photodiode (Thorlabs, DET08CFC/M, bandwidth 5 GHz) and a digital oscilloscope (Agilent Infiniium DSO90604A, bandwidth 6 GHz, 20 GSa/s). The characteristics of the generated DSR pulse were also characterized using an optical spectrum analyzer (Yokogawa, AQ6370B), an electrical spectrum analyzer (Rhode&Schwartz, FPL1007), and a power meter (Thorlabs, PM400 equipped with a S121C photodiode power sensor).

**Fig. 2:**
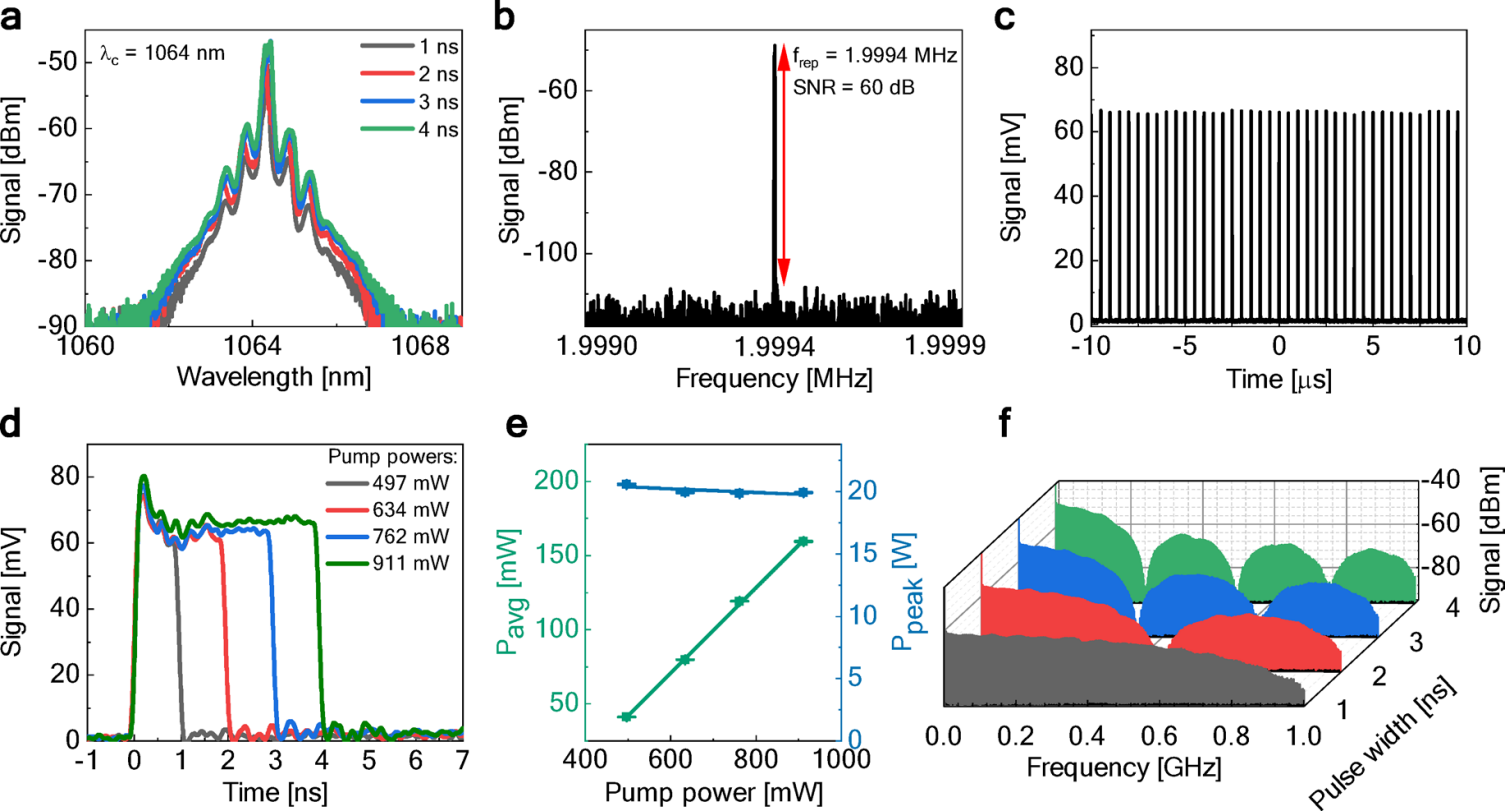
Characterization of the DSR oscillator: (**a**) optical spectra at four pump powers and pulse durations, (**b**) fundamental beat note in the RF spectrum (measured at 1 ns pulse duration), (**c**) pulse train oscilloscope trace (measured at 1 ns pulse duration), (**d**) pulse profiles at four different pump powers showing duration variation from 1 to 4 ns, (**e**) output average and peak powers at different pump powers, (**f**) RF spectral evolution with pump power and pulse duration variations.

Figure 2 shows the characterization of the DSR laser. As with other reported DSR lasers, the pulse duration of the mode-locked rectangular pulses can be easily controlled by varying the resonator gain^28,33^. To evaluate the multiphoton microscopy efficacy, we selected four different pulse durations (1–4 ns) at four different pump powers (497 to 911 mW). We focused our imaging experiments in the 1–4 ns range, where the laser exhibits robust mode-locking and low pulse duration jitter (the laser ceases stable mode-locking at pump powers below ~ 412 mW). Figure 2a shows the optical spectra of the generated pulses. The spectral shape and central wavelength of 1064 nm remained stable across a range of different pump powers and pulse durations. The 3-dB spectral width of all spectra remained below 0.2 nm. Figure 2b and c show that the oscillator operated at a repetition frequency of ~ 2 MHz and maintained a stable pulse train (measured at 1 ns pulse duration). Figure 2d shows that pulse duration increased gradually from 1 to 4 ns with increasing pump power, while pulses retained their rectangular-like shape and peak power. The accuracy of the pulse duration measurements is slightly constrained by the instrument bandwidth, primarily determined by the 5 GHz photodiode (rise time ≈ 70 ps) and the 6 GHz oscilloscope (rise time ≈ 58 ps). The combined system rise time is approximately 91 ps, which is an order of magnitude shorter than the measured pulse durations (1–4 ns). The output average power gradually increased with the increase in pump power, while the output peak power remained approximately the same [Fig. 2e]. Figure 2f presents the oscillator’s radio frequency (RF) spectra for different pulse durations, i.e., pump powers. The spectra exhibited characteristic envelope modulation, which correlates with the pulse duration (also observed in other experiments, e.g.,^34–36^).

**Fig. 3:**
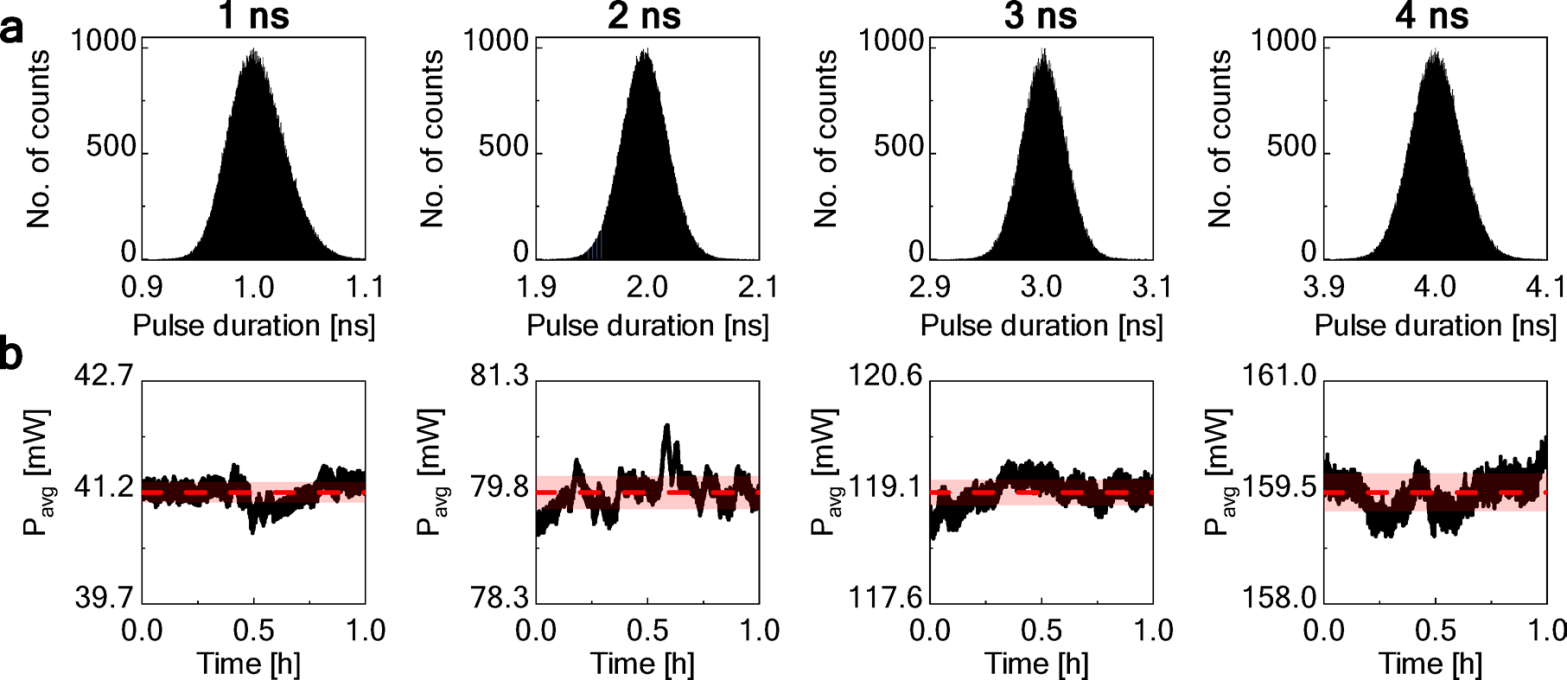
DSR laser stability analysis: (**a**) pulse duration distribution histograms showing pulse duration jitter, (**b**) long-term power stability over time (red dashed line–mean value, shaded area–the standard deviation range).

The pulse duration stability of the generated pulses at four setpoints is further investigated in Fig. 3. Panel (a) presents histograms quantifying the statistical distribution of pulse durations during steady-state operation. This procedure captures both intrinsic laser fluctuations and additive electronic noise, which together result in the observed Gaussian distribution. 1 ns pulses exhibited a mean duration of 1.01 ns with a standard deviation of 74 ps, indicating relatively low pulse duration jitter. The 2 ns configuration showed a mean of 2.03 ns with a 61 ps standard deviation, while 3 ns pulses demonstrated a mean of 3.02 ns with the lowest standard deviation of 57 ps. 4 ns pulses averaged 4.02 ns with a standard deviation of 67 ps. These standard deviation values represent the 3σ jitter. Given the oscilloscope’s 20 GSa/s sampling rate (corresponding to a 50 ps sampling interval), the temporal resolution limited the precision of jitter measurements. As a result, the measured pulse-to-pulse duration jitter likely overestimated the actual fluctuations. Nevertheless, the observed values remain sufficiently low to confirm good temporal stability, which is more than adequate for ensuring consistent illumination in multiphoton imaging applications. Panel (b) presents the corresponding long-term output power stability for each pulse duration setting. The laser’s average output power at each pump power setting was monitored for 1 h. The average output power gradually increased with higher pump power and longer pulse duration. For 1 ns, the mean value of the average power was equal to 41.16 mW (standard deviation of 0.14 mW); for 2 ns, the mean was 79.80 mW (standard deviation of 0.22 mW); for 3 ns, the mean was 119.10 mW (standard deviation of 0.17 mW); and lastly, for 4 ns, the highest average power was observed with a mean of 159.45 mW (standard deviation of 0.25 mW). These small fluctuations in the average power arise from the combined effect of intrinsic laser noise, pump-power variations, and environmental influences associated with the experimental setup (e.g., mechanical vibrations, temperature drifts). These results indicate excellent long-term power stability with all fluctuations remaining well below 1% of the mean power in all cases. Notably, the laser system was not actively optimized for output power stabilization, highlighting the inherent stability of the design under the tested conditions.

### Multiphoton microscope

**Fig. 4:**
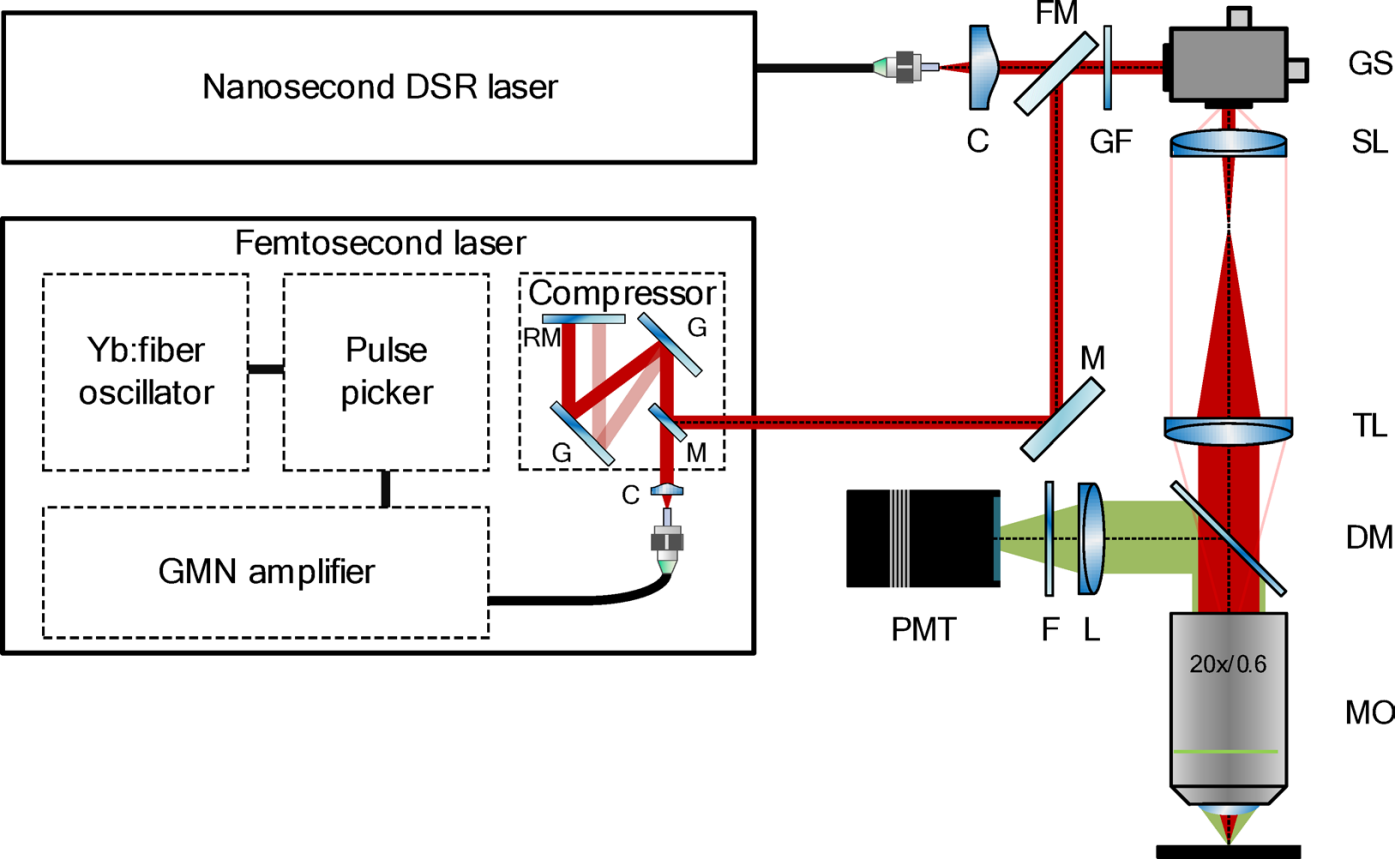
Experimental setup of the multiphoton microscope system. The configuration enabled switching between the nanosecond DSR laser and a femtosecond laser for sample excitation. RM–retro mirror, G–diffraction grating, M–mirror, C–collimator, FM–flip-mirror, GF–gradient index filter, GS–galvanometric scanners, SL–scan lens, TL–tube lens, DM–dichroic mirror, MO–microscope objective, L–lens, F–filter, PMT–photomultiplier tube.

The DSR pulses were outcoupled to free space (via a FC-APC fiber connector), collimated using a collimator (C; Schäfter + Kirchhoff, 60FC-4-A11), and directed towards our home-built multiphoton microscope, working in a two-photon excited fluorescence modality, as seen in Fig. 4.

A gradient index filter (GF; Thorlabs, NDL-25C-2) was used at the entrance of the microscope to adjust the pulse average power delivered to the sample. Subsequently, the laser beam was directed onto a pair of closely coupled galvanometric scanners (ScannerMAX, Saturn-5), followed by transmission through a scan lens (Thorlabs, SL50-2P2) and a tube lens (Thorlabs, TTL200MP) to the microscope objective (Nikon, N20X-PF). Fluorescence emitted from the sample was collected in an epi-detection configuration using the same objective. A dichroic mirror (Semrock, HC 705 LP) and a bandpass filter (Thorlabs, FESH0700; 400–700 nm passband) were used to separate the fluorescence signal from the residual excitation light. The filtered fluorescence was subsequently focused by a lens (Thorlabs, AC254-050-A-ML) onto a photomultiplier tube (Thorlabs, PMT2101/M with a Hamamatsu H10770PA-40 GaAsP photocathode), which was connected to a data acquisition card (NI, PCIe-6363) through a 100 kHz low-pass filter. System control and data acquisition were performed using custom-written software in LabVIEW.

### Image processing

Images were acquired at a resolution of 512 × 512 pixels with a pixel dwell time of 5 µs (FOV of 307.2 µm). For each image, 50 frames were recorded and averaged. Images were plotted in MATLAB using a perceptually-uniform color scale^37^.

### Femtosecond fiber laser

To reliably evaluate the performance of the DSR mode-locked laser in multiphoton microscopy, we compared it with a femtosecond fiber laser (described in detail in^38,39^). The system consisted of a Yb:doped fiber oscillator in a figure-of-eight configuration, a pulse picker allowing for adjustment of the repetition rate, and a gain-managed nonlinear amplifier. Before launching the pulses into the experimental setup, the pulses were compressed in a bulk optic compressor, which was adjusted to pre-compensate the dispersion of the microscope system. The pulses were generated at a central wavelength of ~ 1070 nm and had a duration of 39 fs and an energy of ~ 42 nJ, measured at the sample plane. The pulse picker was used to lower the fundamental repetition frequency of the femtosecond laser (15.2 MHz) to a value comparable with the DSR ML laser—1.9 MHz.

## Results

### Multiphoton imaging performance versus pulse duration at a constant average power

**Fig. 5:**
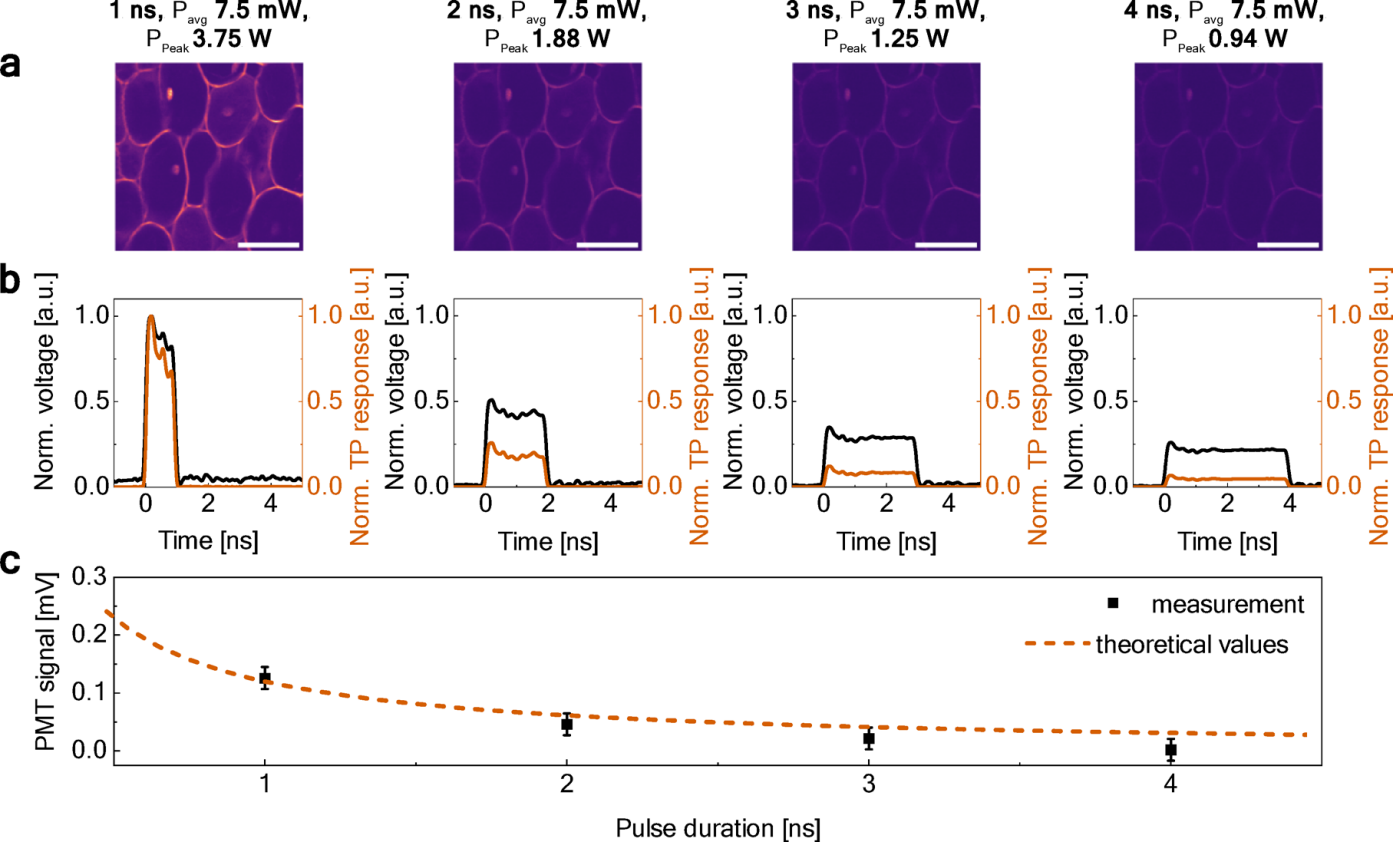
Analysis of pulse duration effects on two-photon imaging at constant repetition rate and average power: (**a**) TPEF images of *convallaria majalis* at increasing pulse durations (scale bars: 100 µm), (**b**) pulse duration (black) and normalized two-photon response (red), (**c**) measured PMT signal from images (points) and theoretical relationship (dashed line).

In the first part of the experiments, we investigated the effect of DSR pulse duration on multiphoton microscopy performance. C*onvallaria majalis* root transverse section stained with an acridine orange was used as a test sample. During the first experiment, we fixed the DSR pulses at an average output power of 7.5 mW at the sample (using the GF filter placed before the microscope) while increasing the pulse duration (the pulse repetition rate was 2 MHz). Results of multiphoton imaging are presented in Fig. 5a for four different pulse durations illustrated in Fig. 5b. The waveforms in Fig. 5b were normalized by scaling their peak amplitudes in proportion to the calculated peak power for each pulse duration, while preserving the integral under each curve to reflect the constant average power across all configurations. Image contrast decreases progressively with longer pulse durations. This trend is consistent with theoretical expectations shown in Eq. (1): increasing the pulse duration at a fixed average power and repetition rate reduces the peak power, which in turn lowers the two-photon excitation efficiency. This is also reflected in the corresponding normalized two-photon (TP) response calculated for each pulse, as a square of instantaneous power [Eq. (2)]. Figure 5c shows this relationship, where the points represent measured PMT signals from the registered images, and the dashed line shows the theoretical prediction for PMT signal versus pulse duration.

### Multiphoton imaging performance versus pulse duration at a constant peak power

**Fig. 6:**
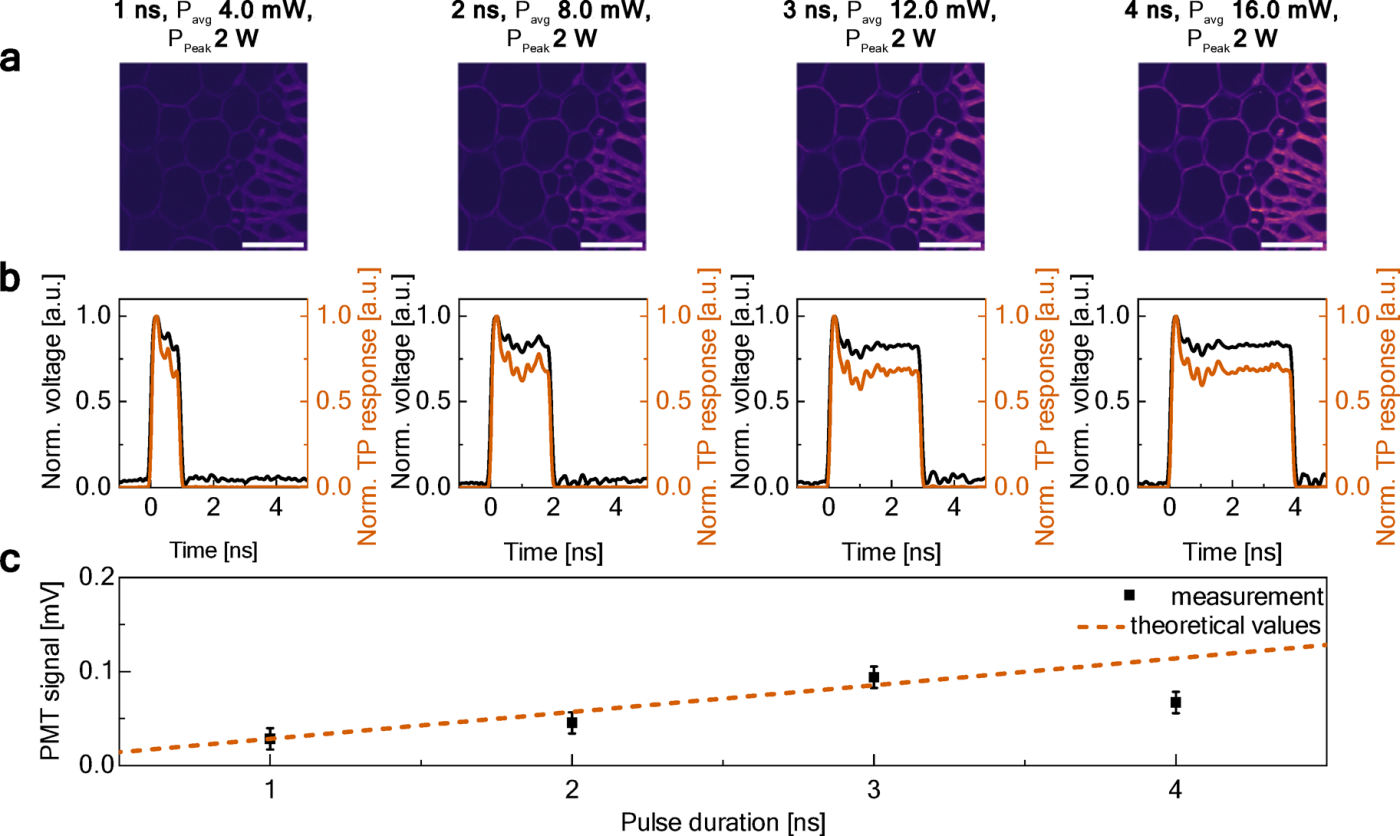
Analysis of pulse duration effects on two-photon imaging at constant repetition rate and peak power: (**a**) TPEF images of *convallaria majalis* at increasing pulse durations (scale bars: 100 µm), (**b**) pulse duration (black) and normalized two-photon response (red), (**c**) measured PMT signal from images (points) and theoretical relationship (dashed line).

In the second experiment, we maintained a constant pulse peak power of the DSR pulses at 2 W and a repetition rate of 2 MHz, while increasing both the pulse duration and the resulting average power at the sample. As in the previous experiment, we used a *convallaria majalis* root sample stained with acridine orange. Figure 6a presents images acquired at various pulse durations, which are detailed in Fig. 6b. Here, the average power increases as a consequence of increasing pulse duration, which, according to Eq. (1), enhances the average number of photons emitted by the fluorescing medium. This trend was reflected in the normalized TP response [shown in red in Fig. 6b] and in the measured PMT voltages shown in Fig. 6c, where the experimental data points follow a linear trend, consistent with the theoretical model (dashed line). The results for 1–3 ns long DSR pulses closely match the theoretical predictions; however, the PMT signal for the 4 ns pulse shows a lower-than-predicted response, likely due to partial PMT saturation in highly fluorescent regions caused by elevated instantaneous photon flux. Findings from both cases highlight a key limitation of using longer pulses in multiphoton microscopy: achieving comparable image quality with longer pulses requires higher average power at the sample, which increases the potential risk of photodamage to delicate biological specimens or may lead to detector saturation. Nevertheless, our results demonstrate that with careful control of acquisition parameters, high-quality images can still be obtained using the non-complex DSR laser, even at longer pulse durations.

### DSR versus femtosecond laser imaging performance

Femtosecond lasers are the standard choice in multiphoton microscopy due to their high peak powers and efficient nonlinear excitation. As presented in Fig. 4, to facilitate direct performance comparison, the experimental setup was designed to acquire fluorescence images using both the DSR laser operating at 1 ns pulse duration and a femtosecond fiber laser emitting 39 fs pulses with a 1.9 MHz repetition rate. We also note that in the case of the femtosecond laser, the use of a precisely aligned bulk-optic pulse compressor was necessary to maintain the duration of the femtosecond pulses at the sample plane.

**Fig. 7:**
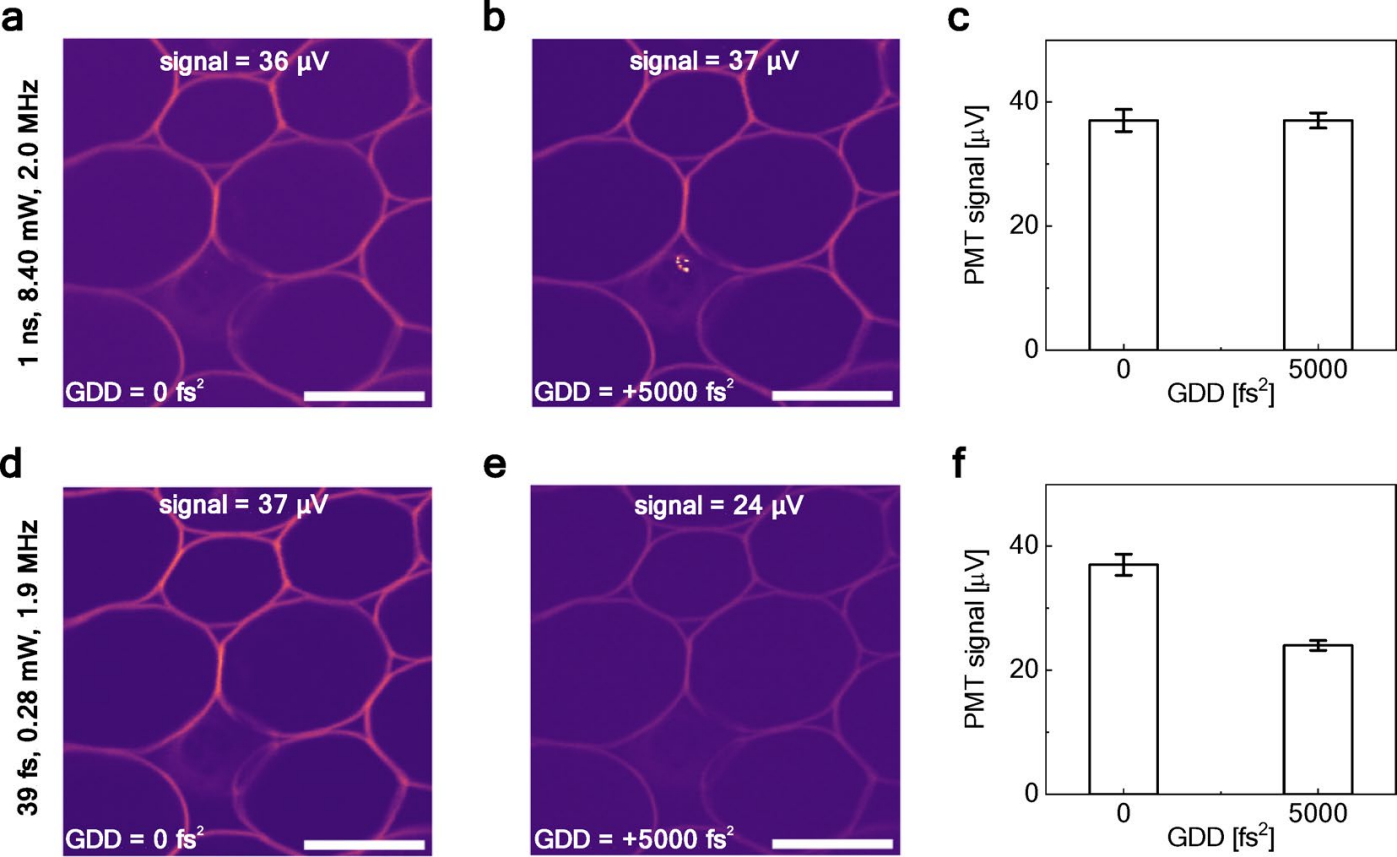
Comparison of TPEF images of *convallaria majalis* obtained in similar conditions using two different lasers. Images obtained using the DSR laser generating 1 ns pulses at the f_rep_ of 2.0 MHz with an average power of 8.4 mW at the sample without (**a**) and with (**b**) added dispersion. (**c**) Corresponding bar chart showing the value of the PMT signal with different GDD values. Images obtained using the femtosecond laser generating 39 fs pulses at the f_rep_ of 1.9 MHz with an average power of 0.28 mW at the sample without (**d**) and with (**e**) added dispersion. (**f**) Corresponding bar chart showing the value of the PMT signal with different GDD values.

We measured the PMT signal obtained using the DSR laser at 8.40 mW average output power at the sample, and we adjusted the average output power of the femtosecond laser accordingly, to match this value of the PMT signal. Results are shown in Fig. 7a and d, respectively. The same average PMT voltage per image as with the DSR laser was received using the 39 fs pulse with an average power of 0.28 mW (see Table 1). Both images display comparable information; however, some minor axial focus shift is visible between the images. This difference is likely due to slight geometric differences in the optical paths of the two beams, which can result in a shift in the effective focal plane at the sample.

**Table 1:** Nanosecond and femtosecond pulse parameters and obtained PMT signal values per image.

${\uptau }_{\text{p}}$	P_avg_	P_peak_	PMT signal
1 ns	8.40 mW	4.20 W	36 µV
39 fs	0.28 mW	3.78 kW	37 µV

In addition to the overall imaging performance, Fig. 7 also illustrates a key distinction in the behavior of the two laser sources under the influence of group delay dispersion (GDD). Dispersion was added to the system by placing two dispersive glass blocks (optical glass H-ZF13, width of 20 mm) in the beam’s path. For the DSR laser, the acquired images [panels (a) and (b)] remain very similar even after the introduction of a large amount of GDD (+ 5000 fs^2^, which is an equivalent to exchanging a low NA objective for a high NA one^40^), and the corresponding bar chart in Fig. 7c confirms that the detected signal is unaffected, maintaining the PMT signal at around 40 µV. In contrast, the femtosecond laser images [panels (d) and (e)] show a visible reduction in brightness under the same conditions, with the bar chart in Fig. 7f further quantifying the decrease in the detected signal from 37 to 24 µV. This comparison highlights an important practical advantage of the DSR source: its insensitivity to dispersion eliminates the need for precise pre-compensation optics, which are otherwise essential for femtosecond imaging. As a consequence, the DSR laser allows the introduction of additional optical elements into the beam path without a significant impact on imaging performance, while for femtosecond excitation, each optical element may introduce dispersion that degrades signal strength. This robustness simplifies system operation and alignment, while still enabling high-quality multiphoton imaging.

Despite these small differences, both laser sources yielded high-quality and informative images. However, although the average power at the sample was significantly higher for the nanosecond DSR laser when compared to the femtosecond one, the peak power of the femtosecond source was higher due to the shorter pulse duration. Having 1 ns pulses at a 2 MHz repetition rate for the DSR laser, the peak power was approximately 4.20 W. For the femtosecond laser, with 39 fs pulses at 1.9 MHz repetition rate, the estimated peak power reached 3.78 kW (Table 1). This tradeoff between peak and average power has practical implications for imaging delicate samples^41^. The femtosecond laser, with its high peak power and low average power, enables efficient multiphoton excitation with minimal thermal load, making it especially suitable for imaging delicate biological tissues^42,43^. This is particularly advantageous in applications where thermal damage must be minimized, such as tissues containing highly absorbing chromophores like melanin, commonly found in the skin or eye tissue, when single-photon absorption in the near-infrared can lead to photothermal damage^15,44^. In contrast, the nanosecond DSR laser relies on higher average power to achieve a sufficient photoluminescence signal, which could introduce some photothermal effects over extended imaging sessions in the case of delicate samples^27,44^. However, nanosecond pulses have lower peak power, which may help reduce the nonlinear photodamage caused by multiphoton ionization or free electron generation^45^. This can be beneficial in applications prioritizing robust, long-term imaging of more resilient tissues or where broader excitation and simpler laser systems are desirable^46^. Therefore, sample sensitivity should be a key consideration when selecting the excitation source. However, it is important to note that the power levels used in our experiments with nanosecond pulses were within typical ranges for multiphoton imaging.

### Evaluation of fiber delivery for endoscopic applications

Fiber delivery is often required for multiphoton endoscopy and other in vivo applications. However, femtosecond pulses are highly sensitive to chromatic dispersion and nonlinear effects that might occur during transmission through fibers, which can degrade image quality unless carefully compensated. To evaluate the performance of the nanosecond DSR source under such conditions, we compared imaging with direct free-space delivery and with an additional 3 m of spliced single-mode fiber (PM980-XP). Images were obtained using DSR-generated 1 ns pulses with an average power of 7.65 mW at the sample plane.

**Fig. 8:**
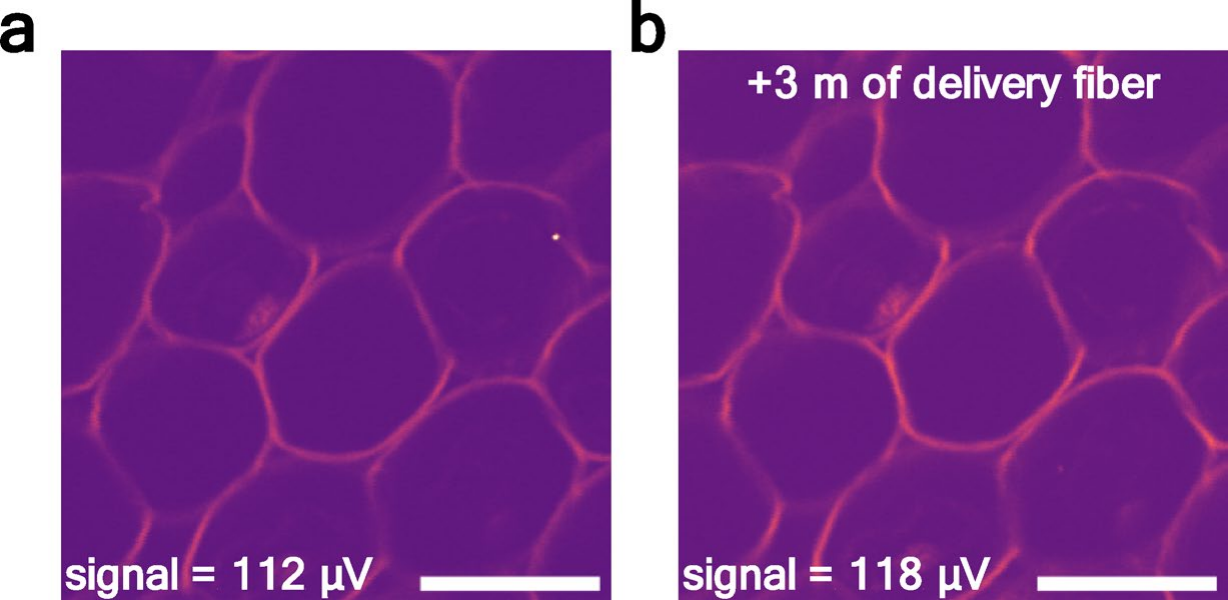
Comparison of TPEF images of *convallaria majalis* obtained using the DSR laser generating 1 ns pulses with the average power of 7.65 mW at the sample: (**a**) directly from the laser’s output, and (**b**) with an additional 3 m of fiber. Scale bars: 100 µm.

Multiphoton images acquired in these two configurations are presented in Fig. 8a and b (without and with added fiber, respectively). Quantitative analysis of the PMT signal revealed mean values of 112 µV without the added fiber and 118 µV with 3 m of fiber, indicating no measurable degradation of the imaging performance.

These results confirm that the nanosecond DSR pulses are resilient to chromatic dispersion and fiber-induced nonlinearities over typical endoscopic delivery lengths^47^. These findings support the suitability of DSR nanosecond lasers for fiber-based applications, where their insensitivity to dispersion simplifies system integration. By removing the need for complex pre-compensation, this offers a robust and practical pathway toward compact, low-cost, and flexible endoscopic microscopy.

## Discussion

This work presents a low-cost, all-fiber DSR laser-based imaging setup^28^ and demonstrates its applicability for multiphoton microscopy. The implementation of a DSR laser as the excitation source provides several practical advantages: it is inexpensive to build (estimated material cost at a 1500$ level, assuming individual unit prices; bulk purchasing may further reduce costs), mechanically robust due to its all-in-fiber layout, and easy to construct, making it an accessible alternative to more complex ultrafast fiber lasers. The fully PM architecture also contributes to improved power stability, ensuring consistent excitation conditions during imaging. Additionally, nanosecond pulses have a much lower sensitivity to dispersive effects. This makes the DSR laser particularly attractive for fiber-delivered or portable imaging setups, including potential use in endoscopic applications^30^, where maintaining pulse integrity over long fiber-based delivery optics is critical.

Multiphoton microscopy is conventionally performed using ultrashort femtosecond pulses, which offer high peak powers and precise spatial localization of excitation. Nonetheless, we have shown that nanosecond pulses can also be used in effective multiphoton excitation. In this work, we investigated pulse durations of 1 ns, 2 ns, 3 ns, and 4 ns while maintaining constant average power at the sample. As expected, the fluorescence signal decreased with increasing pulse duration due to the inverse correlation between pulse duration and peak power—when the average power is held constant, longer pulses inherently result in lower peak intensities, reducing two-photon excitation efficiency.

Notably, the DSR laser system maintains the peak power even as the pump power, and consequently the pulse duration, increases^28,33^. This enables a tunability of the system in which it is possible to increase average power at the sample by increasing the pulse duration without a corresponding change in peak power. However, achieving comparable image quality with longer pulses does require higher average power at the sample, which may increase the risk of photodamage to delicate biological specimens or lead to photodetector saturation^27,44^. Nevertheless, our findings demonstrate that, with careful adjustment of acquisition parameters, high-quality images can still be achieved using the DSR laser, even at extended pulse durations. This tunability is especially advantageous for imaging samples with varying sensitivity to laser exposure or with differing imaging depth requirements, allowing users to fine-tune excitation conditions while managing potential damage.

We also performed a comparative analysis between the DSR laser and a standard femtosecond laser^38^ delivering 39 fs pulses at a 1.9 MHz repetition rate. Despite using a significantly higher average power at the sample (8.40 mW vs. 0.28 mW), the DSR laser’s peak power (4.20 W) was considerably lower than that of the femtosecond source (3.78 kW). This trade-off between the average and peak power shows the difference in potential applications. Femtosecond sources are ideal for sensitive samples^42,43^ (e.g., skin and ophthalmic imaging, where one-photon absorption of infrared light by melanin granules can cause thermal damage to the sample^15,44^), offering high excitation efficiency with minimal thermal impact. In contrast, the nanosecond DSR laser may be more appropriate for more resilient samples, where higher average power can be used without causing any damage^46^. Moreover, its lower peak intensity may help reduce the likelihood of nonlinear photodamage mechanisms, such as optical breakdown or plasma generation, which are typically associated with ultrashort, high-intensity pulses^45^.

Similar to other fiber lasers, a DSR laser operates in the 1064 nm spectral range. This restricts compatibility with many fluorophores optimized for excitation in the 700–900 nm range. However, in recent years, many probes and fluorophores have been developed for longer wavelength excitation to take advantage of deeper penetration depths. Examples include Alexa Fluor 568 and 594, mKate2, mOrange2, mRuby2, PY-1268, and many others^48,49^. The 1064 nm wavelength is also perfectly suited for SHG, as the 532 nm wavelength coincides with the peak of sensitivity of many common photomultipliers. Nonlinear frequency conversion techniques of DSR lasers were also investigated, such as SHG and supercontinuum, demonstrating potential for broader spectral coverage^50,51^.

To our knowledge, this is the first demonstration of a simple, all-fiber DSR laser applied successfully to two-photon microscopy. Its performance, combined with system simplicity and cost-effectiveness, suggests that DSR lasers represent a promising direction for expanding access to nonlinear imaging techniques. This study opens the door for further exploration of alternative laser architectures in multiphoton systems, particularly for applications where flexibility, affordability, and robustness are essential.

## Data Availability

Data underlying the results presented in this paper are available in Ref^52^.
